# The impact of COVID-19 lockdowns on primary care contact among vulnerable populations in England: a controlled interrupted time-series study

**DOI:** 10.3399/BJGPO.2025.0017

**Published:** 2025-12-19

**Authors:** Scott R Walter, Chris Salisbury, Lauren J Scott, Frank de Vocht, John Macleod, Yoav Ben-Shlomo, Helen J Curtis, Aziz Sheikh, Srinivasa V Katikireddi, Amir Mehrkar, Sebastian Bacon, George Hickman, Ben Goldacre, The OpenSAFELY Collective, Maria Theresa Redaniel

**Affiliations:** 1 National Institute for Health and Care Research, Applied Research Collaboration West (NIHR ARC West), University Hospitals Bristol and Weston NHS Foundation Trust, Bristol, UK; 2 Population Health Sciences, Bristol Medical School, University of Bristol, Bristol, UK; 3 Centre for Academic Primary Care, Bristol Medical School, University of Bristol, Bristol, UK; 4 Bennett Institute for Applied Data Science, Nuffield Department of Primary Care, University of Oxford, Oxford, UK; 5 Nuffield Department of Primary Care, University of Oxford, Oxford, UK; 6 MRC/CSO Social & Public Health Sciences Unit, University of Glasgow, Glasgow, UK

**Keywords:** COVID-19, primary health care, vulnerable patients, general practitioners, primary healthcare

## Abstract

**Background:**

UK COVID-19 lockdowns significantly affected primary care access and delivery. Little is known about whether lockdowns disproportionally impacted vulnerable groups, including people who misuse substances, people who have experienced domestic violence or abuse, those with intellectual disability, and children with safeguarding concerns.

**Aim:**

To evaluate the impact of UK COVID-19 lockdowns on primary care contact rates among vulnerable groups.

**Design & setting:**

Natural experimental design using all registered patients in the OpenSAFELY platform.

**Method:**

With approval from NHS England, we conducted controlled interrupted time-series analyses on records from 24 million patients in England between September 2019 and September 2021.

**Results:**

Pre-pandemic, primary care contact rates were 110.1 per 1000 patients per week. Following the initiation of the first lockdown (23 March 2020), there was a large reduction of 29–61 contacts per 1000 patients per week among vulnerable and general population groups. For patients with alcohol misuse, those aged ≥14 years with intellectual disability, and children with safeguarding concerns, this reduction was significantly more extreme than corresponding general populations (relative rate difference -23.8 [95% confidence interval {CI} = -39.8 to -7.7, *P* = 0.003], -24.6 [95% CI = -38.8 to -10.5, *P*<0.001], and -15.4 [95% CI = -26.9 to -3.8, *P* = 0.009], respectively). Following the final lockdown (29 March 2021), all groups had contact rates exceeding pre-pandemic rates (with increases more marked in vulnerable populations), except those only including children.

**Conclusion:**

Our results suggested a larger short-term impact of the first COVID-19 lockdown on primary care contact for some vulnerable groups, compared with the general population, and differential impacts persisted through subsequent lockdowns and beyond for some vulnerable groups. There is a need to examine drivers of these differences to enable more equitable primary care access and provision.

## How this fits in

There is limited knowledge on how the COVID-19 pandemic and resultant lockdowns affected primary care contact for vulnerable people. We used primary care records from 24 million patients in England to evaluate the impacts of the UK COVID-19 lockdowns on primary care service use in vulnerable groups, including patients with issues of substance misuse or dependence, those who had experienced domestic violence or abuse, patients with intellectual disability, and children with safeguarding concerns, compared with the rest of the population. Following the initiation of the first lockdown (23 March 2020), there was a large reduction in primary care contacts for all vulnerable and non-vulnerable patient groups. For patients with alcohol misuse issues, those aged ≥14 years with intellectual disability, and children with safeguarding concerns, this reduction was significantly larger than the corresponding general population. Differential effects persisted through subsequent lockdowns. It is important to understand the drivers of the observed differences to enable more equitable primary care access and provision, both now and in the event of a future pandemic or large-scale change in availability of primary care for other reasons.

## Introduction

The impact of the COVID-19 pandemic has been profound and far-reaching, with around 70% of people in England infected and more than 200 000 COVID-19-related deaths.^1–3^ The inequity of those impacts has been well documented, where those with pre-existing morbidities or disabilities, and people from certain ethnic minorities, regions or age groups were disproportionately affected.^4^

The lockdowns in response to the pandemic in the UK dramatically changed health service provision and use. There is widespread evidence of reduced healthcare access at the outset of the first national UK lockdown, including a decrease in hospital admission rates, particularly cancer admissions,^5,6^ and accident and emergency presentations.^7–9^

In the initial lockdown, there was an accelerated shift towards remote primary care consultations,^10^ as well as an overall decrease in the number of consultations.^10–13^ Differential decreases in primary care contacts have been observed for patients depending on their condition, with diabetic emergency and depression among the most affected.^12^ Foley *et al* ^11^ reported a 41% reduction in contacts among children and young people. In contrast, people with poor mental health, those who were shielding, and those aged ≥85 years had similar or higher consultation rates through the initial lockdown period compared with the same period the previous year.^10^ A potential knock-on effect of reduced consulting is a decline in rates of diagnoses for physical and mental health conditions.^13^

On average, the general population has around five primary care appointments per year.^14–16^ However, those in vulnerable groups often have higher contact (for example, people who misuse substances often have higher rates of physical^17^ and mental^18^ health disorders than those who do not misuse substances, and people who use illicit drugs may require regular contact with primary care to be issued substitute drug prescriptions). There is little evidence on how the pandemic and resultant lockdowns affected primary care contact in vulnerable groups.

This study aimed to assess changes in primary care contact rates in England through changing lockdown states among vulnerable populations, including people who misuse substances, people who experience(d) domestic violence or abuse (DVA), those with intellectual disability, and children with safeguarding concerns, relative to the general population.

## Method

### Study design

This study used a controlled interrupted time-series (CITS) design applied to a cohort of approximately 24 million registered patients (approximately 40% of the population)^19^ from English primary care practices that use TPP SystmOne software.

Eight vulnerable populations were studied: patients with alcohol misuse; patients with drug misuse; those with opioid dependence; females who had experienced DVA; males who had experienced DVA; people with intellectual disability aged <14 years; those with intellectual disability aged ≥14 years; and children (aged <18 years) with safeguarding concerns. These populations were chosen as they represent groups that have not been previously studied in this context and who were possible to identify using codelists.

**Figure 1.**
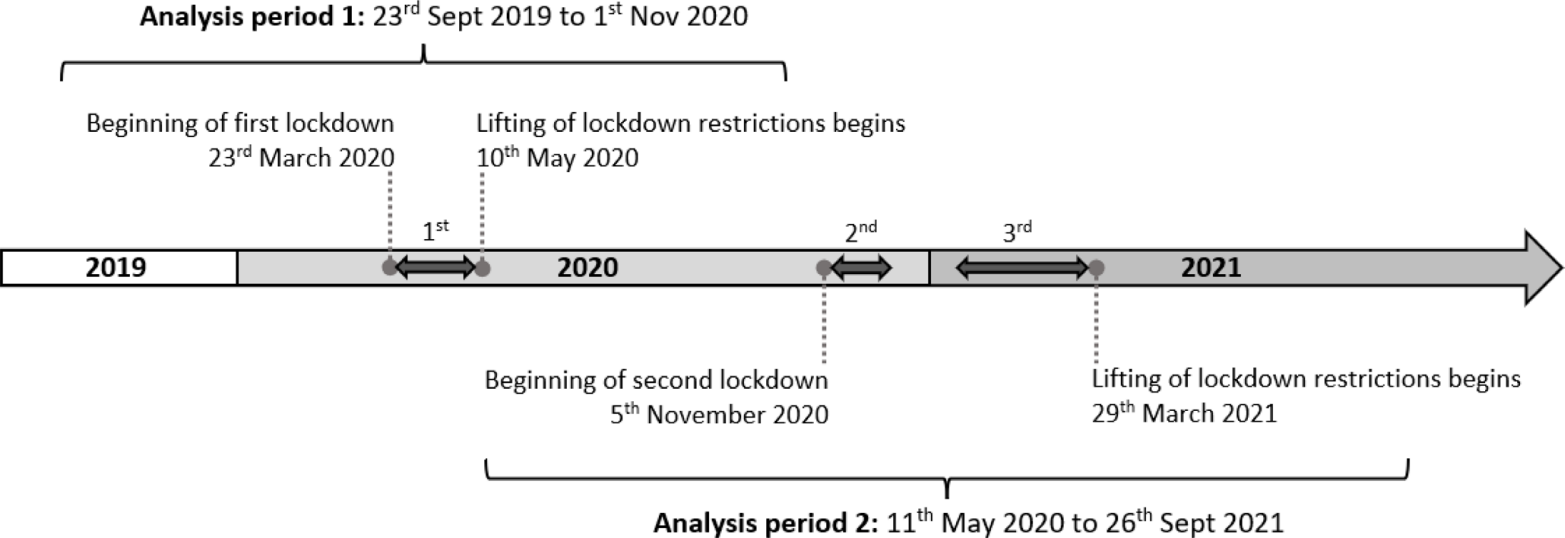
Timeline of UK national COVID-19 lockdowns and the analysis periods used in this study

Two sets of analyses were performed exploring the first UK COVID-19 lockdown (23 March 2020–09 May 2020),^20^ and the second and third UK lockdowns combined as a single period (05 November 2020–28 March 2021).^21,22^ Both analyses included 6-months of data before and after these dates (Figure 1; some control periods were truncated by a few days so as not to include active lockdown periods).

### Data source

The OpenSAFELY platform was established to support urgent research into the COVID-19 emergency with the Control of Patient Information (COPI) notice forming the legal basis for its establishment.^23^ All data were linked, stored and analysed securely within the OpenSAFELY platform.^24^ Data include pseudonymised data such as coded diagnoses, medications, and physiological parameters. All code is shared openly for review and re-use under an MIT open licence.^25^

### Population

Codelists were used to identify vulnerable populations^25^ (Supplementary material 1). For a given codelist, any registered patient with a matching code in their primary care record during a 1-year lookback period (this was a moving period, with the cohort of interest updated each week) was classified as belonging to that vulnerable population, while all patients without that vulnerability formed the general comparison population. For vulnerable groups defined by age or sex, the comparison population were those within the same age or sex groups but without the relevant vulnerability.

### Outcome

The primary outcome was the rate of all primary care contacts per 1000 patients per week, constructed as the weekly count of contacts divided by the number of registered patients at the beginning of each week. The data were derived from the TPP appointments table, which is not necessarily an exact record of face-to-face or virtual consultations, but is considered a better approximation than the alternative of deriving data from consultation tables.^26^

### Statistical methods

Within a CITS framework, linear regression models analysed each of the eight vulnerable groups and two analysis periods (16 models in total). The models estimated a step change (the immediate change) and slope change (the average weekly change) in the vulnerable and general populations, at the start and end of the lockdown periods (Supplementary material 2 & 3). Results are presented as contact rate differences (RDs) per 1000 patients and their corresponding 95% confidence intervals (CIs) and *P* values for the vulnerable populations, the comparative general populations, and the relative RD between the two.

Models were adjusted for public holidays since contact rates were markedly lower during these periods. Seasonal effects were not accounted for since there was little evidence of seasonal change in the outcome (in sensitivity analyses, including calendar month had little impact on estimates). Although there may be differences in factors, such as age distribution and deprivation between vulnerable and general populations, these are reasonably stable over time so would not impact estimates of change when modelling weekly aggregated data.

Analyses were performed using Python (version 3.9) and Stata MP (version 17); all analysis codes are recorded on Github.^25^

## Results

### Rates of primary care contact before the first UK lockdown

In the pre-lockdown period, the crude primary care contact rate in the general population was 110.1 per 1000 patients per week; women were more likely to have contact with their GP than men, and adults more likely than children (Table 1).

**Table 1.** Crude rates of primary care contact per registered 1000 patients per week, by vulnerable group and time period

Group	Period	Substance misuse			Domestic violence or abuse		Intellectual disability		Safeguarding
		**Alcohol misuse**	**Drug misuse**	**Opioid dependence**	**Female**	**Male**	**≥14 years**	**<14 years**	**<18 years**
**Vulnerable population**	Pre-lockdown: Sep 2019–Mar 2020	220.9	283.5	311.3	251.4	118.4	202.9	88.0	63.0
	1st lockdown: Mar 2020–May 2020	162.9	258.0	305.9	214.6	86.7	146.3	49.4	31.9
	Between lockdowns: May 2020–Nov 2020	226.3	310.1	347.5	267.1	108.5	202.7	63.7	43.5
	2nd & 3rd lockdowns: Nov 2020–Mar 2021	262.2	347.6	385.7	287.0	121.2	246.8	72.6	45.7
	Post-lockdowns: Mar 2021–Sep 2021	256.2	335.3	376.6	281.6	128.2	213.9	82.5	55.9
	Total	233.2	310.8	346.1	266.7	117.0	209.7	75.7	51.3
	% of patient population	0.17	0.07	0.37	0.18	0.06	0.59	0.44	2.17
	1 patient per *n* population	606	1429	271	554	1610	169	228	46
**General population^a^**	Pre-lockdown: Sep 2019–Mar 2020	109.7	109.8	110.0	131.1	88.9	118.0	66.3	105.8
	1st lockdown: Mar 2020–May 2020	70.5	70.5	70.7	85.9	55.5	77.4	34.2	59.7
	Between lockdowns: May 2020–Nov 2020	99.0	99.1	99.3	121.3	77.4	109.5	43.9	75.2
	2nd & 3rd lockdowns: Nov 2020–Mar 2021	110.6	110.7	111.0	134.8	87.2	122.7	45.5	83.8
	Post-lockdowns: Mar 2021–Sep 2021	114.1	114.3	114.5	138.7	90.4	124.6	57.5	97.8
	Total	105.7	105.8	106.0	128.1	83.9	115.6	52.8	89.4

^a^Rates relate to the non-vulnerable population, for example, the general population rate in the ‘female DVA’ column represents females without a history of DVA. DVA = domestic violence or abuse.

Rates of primary care contact throughout the study period were generally higher among vulnerable groups compared with the rest of the population (Table 1). In particular, contact rates for patients with substance misuse, female patients with experience of DVA, and patients aged ≥14 years with intellectual disability were approximately 2–3 times higher than their comparative general populations. In contrast, children with safeguarding concerns had about half the number of primary care contacts compared with children who had no record of safeguarding concerns.

Following the final lockdown, primary care contact rates were slightly higher than pre-pandemic rates in most vulnerable and general population groups, but patients with substance misuse issues and females who experienced DVA had considerably higher contact rates than before the first lockdown (Table 1). For patients aged <14 years with intellectual disability and children with safeguarding concerns, post-lockdown contact rates remained lower than pre-lockdown rates, as did the rates in their corresponding general populations (Table 1).

### Controlled interrupted time-series analysis

#### Analysis period one: first UK lockdown (23 September 2019–1 November 2020)

When the UK first locked down, there was a large reduction in primary care contacts of 29–61 contacts per 1000 patients per week among vulnerable groups and 29–39 contacts per 1000 patients in the general population (step change 1 in Table 2). For patients with alcohol misuse issues, those with intellectual disability aged ≥14 years, and children with safeguarding concerns, the decrease was more significant compared with general populations (relative RD -23.8 [95% CI = -39.8 to -7.7, *P* = 0.003], -24.6 [95% CI = -38.8 to -10.5, *P*<0.001], and -15.4 [95% CI = -26.9 to -3.8, *P* = 0.009], respectively; step change 1 in Table 2).

**Table 2.** Rate difference estimates (per 1000 patients) of step and slope changes from controlled interrupted time-series modelling for the period of the first national UK lockdown

	Vulnerable population		General population		Vulnerable versus general	
	RD (95% CI)	*P* value	RD (95% CI)	*P* value	Relative RD (95% CI)	*P* value
**Alcohol misuse**						
Step change 1	-58.1 (-72.7 to -43.5)	<0.001	-34.4 (-43.2 to -25.5)	<0.001	-23.8 (-39.8 to -7.7)	0.003
Slope change 1	5.6 (2.9 to 8.2)	<0.001	1.8 (-0.2 to 3.9)	0.08	3.7 (0.5 to 7.0)	0.02
Step change 2	5.5 (-4.9 to 15.9)	0.30	-4.1 (-16.0 to 7.7)	0.49	9.6 (-5.8 to 25.1)	0.22
Slope change 2	-2.0 (-4.7 to 0.8)	0.16	0.3 (-1.8 to 2.3)	0.81	-2.2 (-5.5 to 1.1)	0.17
**Drug misuse**						
Step change 1	-48.1 (-62.0 to -34.3)	<0.001	-35.2 (-45.2 to -25.2)	<0.001	-12.9 (-29.6 to 3.8)	0.13
Slope change 1	6.9 (4.2 to 9.6)	<0.001	2.1 (-0.5 to 4.7)	0.12	4.9 (1.2 to 8.5)	0.009
Step change 2	1.1 (-13.2 to 15.4)	0.88	-5.3 (-20.7 to 10.1)	0.49	6.4 (-13.9 to 26.7)	0.54
Slope change 2	-5.7 (-8.4 to -2.9)	<0.001	-0.0 (-2.6 to 2.6)	0.98	-5.6 (-9.4 to -1.9)	0.003
**Opioid dependence**						
Step change 1	-29.2 (-42.9 to -15.5)	<0.001	-34.6 (-45.6 to -23.7)	<0.001	5.5 (-12.3 to 23.3)	0.55
Slope change 1	6.8 (4.4 to 9.3)	<0.001	2.0 (-0.8 to 4.8)	0.15	4.8 (1.2 to 8.4)	0.008
Step change 2	-5.1 (-18.4 to 8.3)	0.45	-5.8 (-22.0 to 10.5)	0.48	0.7 (-19.8 to 21.2)	0.94
Slope change 2	-6.0 (-8.6 to -3.5)	<0.001	0.0 (-2.8 to 2.8)	0.99	-6.0 (-9.7 to -2.4)	0.001
**Domestic violence and abuse**						
***Females***						
Step change 1	-53.3 (-72.9 to -33.8)	<0.001	-39.4 (-50.3 to -28.6)	<0.001	-13.9 (-25.0 to 7.1)	0.19
Slope change 1	4.9 (1.6 to 8.1)	0.003	2.2 (-0.3 to 4.6)	0.09	2.7 (-1.2 to 6.6)	0.18
Step change 2	9.6 (-4.6 to 23.8)	0.18	-4.3 (-18.1 to 9.5)	0.54	14.0 (-5.4 to 33.3)	0.16
Slope change 2	-3.9 (-7.2 to -0.7)	0.02	0.29 (-2.20 to 2.8)	0.82	-4.2 (-8.2 to -0.2)	0.02
***Males***						
Step change 1	-31.1 (-36.6 to -25.6)	<0.001	-28.9 (-35.0 to -22.8)	<0.001	-2.3 (-10.1 to 5.6)	0.57
Slope change 1	-1.0 (-1.6 to -0.4)	0.001	1.0 (0.0 to 2.0)	0.05	-2.0 (-3.1 to -0.9)	<0.001
Step change 2	9.7 (4.1 to 15.3)	0.001	-1.8 (-6.5 to 2.9)	0.45	11.5 (4.2 to 18.9)	0.002
Slope change 2	1.6 (0.9 to 2.3)	<0.001	0.8 (-0.3 to 1.8)	0.15	0.9 (-0.3 to 2.1)	0.15
**Intellectual disability**						
***Aged <14 years***						
Step change 1	-37.0 (-45.7 to -28.4)	<0.001	-32.9 (-39.7 to -26.1)	<0.001	-4.1 (-14.6 to 6.3)	0.44
Slope change 1	-0.5 (-1.7 to 0.8)	0.46	0.2 (-0.7 to 1.1)	0.68	-0.7 (-2.1 to 0.8)	0.36
Step change 2	-6.3 (-11.9 to -0.6)	0.03	-5.7 (-9.9 to -1.5)	0.008	-0.6 (-7.6 to 6.5)	0.88
Slope change 2	2.1 (0.9 to 3.3)	<0.001	0.8 (-0.1 to 1.6)	0.07	1.3 (-0.0 to 2.6)	0.06
***Aged* ≥*14 years***						
Step change 1	-61.1 (-72.8 to -49.4)	<0.001	-36.4 (-45.2 to -27.7)	<0.001	-24.6 (-38.8 to -10.5)	<0.001
Slope change 1	5.4 (3.9 to 6.8)	<0.001	2.2 (0.1 to 4.3)	0.04	3.2 (0.7 to 5.6)	0.01
Step change 2	-10.6 (-20.3 to -1.0)	0.03	-2.5 (-13.8 to 8.7)	0.66	-8.1 (-22.8 to 6.6)	0.28
Slope change 2	-1.2 (-2.8 to 0.5)	0.16	0.10 (-2.0 to 2.2)	0.92	-1.3 (-3.8 to 1.3)	0.32
**Children with safeguarding concerns**						
Step change 1	-47.8 (-58.2 to -37.3)	<0.001	-32.4 (-39.0 to -25.8)	<0.001	-15.4 (-26.9 to -3.8)	0.009
Slope change 1	0.4 (-1.1 to 1.9)	0.59	0.3 (-0.6 to 1.2)	0.50	0.11 (-1.5 to 1.7)	0.90
Step change 2	-5.7 (-10.9 to -0.6)	0.03	-4.8 (-9.2 to -0.4)	0.03	-0.94 (-7.7 to 5.8)	0.79
Slope change 2	1.0 (-0.5 to 2.5)	0.21	0.7 (-0.2 to 1.5)	0.11	0.3 (-1.3 to 1.9)	0.74

Step change 1: change in GP contact rate at the start of the first lockdown (23 March 2020). Slope change 1: change in slope of GP contact rate trend between first lockdown period (23 March 2020–10 May 2020) versus the pre-lockdown period (19 September 2019–22 March 2020). Step change 2: change in GP contact rate at the end of the first lockdown (11 May 2020). Slope change 2: change in slope of GP contact rate trend between the post-lockdown period (11 May 2020–01 November 2020) versus the lockdown period (23 March 2020–10 May 2020). RD = rate difference

Following the initial sharp decrease, contact rates for most groups gradually rebounded through the first lockdown. Rates for those with alcohol misuse, drug misuse, opioid dependence, and those aged ≥14 years with intellectual disability increased significantly more throughout the first lockdown than their general population comparators (relative weekly change in RD 3.7 [95% CI = 0.5 to 7.0, *P* = 0.02], 4.9 [95% CI = 1.2 to 8.5, *P* = 0.009], 4.8 [95% CI = 1.2 to 8.4, *P* = 0.008], and 3.2 [95% CI = 0.7 to 5.6, *P* = 0.01], respectively; slope change 1 in Table 2), with contact rates for patients in all substance misuse categories exceeding pre-COVID rates following the first lockdown (Table 1). However, for males experiencing DVA, this rebound happened a little later following the end of the first lockdown (Table 1 and slope and step change 2 in Table 2). The rebound effect was less apparent for children, where there was a further decrease in contact rates at the end of the lockdown period (for children with safeguarding concerns, intellectual disability aged <14 years, and their corresponding general population comparators), but no significant difference between vulnerable patients and the general population were seen (step change 2 in Table 2).

#### Analysis period two: second and third UK lockdowns (11 May 2020–26 September 2021)

There was no evidence of a step change at the start of the second lockdown in any group. In drug misuse, opioid dependence, and female DVA groups, there was a significant increasing trend in contact rate during the period when the second and third lockdowns were in effect, compared with the general population (relative weekly chainge in RD 2.5 [95%CI = 1.1 to 3.9, *P*<0.001], 3.9 [95% CI = 2.4 to 5.4, *P*<0.001], and 2.0 [95% CI = 0.8 to 3.1, *P* = 0.001], respectively; slope change 3 in Table 3). At the end of the final lockdown, a step decrease was observed among alcohol misuse, drug misuse, opioid dependence, female DVA, and those aged ≥14 years with intellectual disability, in comparison to a step increase in the general population (relative RD -20.7 [95%CI = -34.4 to -7.0, *P* = 0.003], -33.9 [95% CI = -51.4 to -16.4, *P*<0.001], -41.3 [95% CI = -57.6 to -25.1, *P*<0.001], -23.2 [95% CI = -38.4 to -8.0, *P* = 0.003], and -40.4 [95% CI = -59.9 to -20.9, *P*<0.001], respectively; step change 4 in Table 3). For three of these groups — drug misuse, opioid dependence, and female DVA — this was followed by a final slope decrease over and above their corresponding general populations following restrictions easing after the final lockdown (relative weekly change inRD -3.1 [95%CI = -4.6 to -1.6, *P*<0.001], -4.1 [95% CI = -5.6 to -2.5, *P*<0.001], and -1.6 [95% CI = -2.8 to 0.4, *P* = 0.01], respectively; slope change 4 in Table 3).

**Table 3.** Rate difference estimates (per 1000 patients) of step and slope changes from controlled interrupted time-series modelling for the period of the combined second and third national UK lockdowns

	Vulnerable population		General population		Vulnerable versus general	
	RD (95% CI)	*P* value	RD (95% CI)	*P* value	Relative RD (95% CI)	*P* value
**Alcohol misuse**						
Step change 3	-10.6 (-22.3 to 1.0)	0.07	-1.9 (-11.0 to 7.1)	0.68	-8.7 (-25.0 to 7.62)	0.30
Slope change 3	-0.7 (-1.4 to 0.0)	0.05	-1.5 (-2.0 to -0.9)	<0.001	0.8 (-0.2 to 1.8)	0.10
Step change 4	-7.9 (-19.9 to 4.2)	0.20	12.8 (4.7 to 20.9)	0.002	-20.7 (-34.4 to -7.0)	0.003
Slope change 4	-3.4 (-4.4 to -2.5)	<0.001	-0.9 (-1.5 to -0.2)	0.01	-2.6 (-3.8 to -1.4)	<0.001
**Drug misuse**						
Step change 3	1.3 (-14.4 to 16.9)	0.88	0.7 (-11.1 to 12.6)	0.90	0.5 (-21.6 to 22.6)	0.96
Slope change 3	0.9 (-0.2 to 2.0)	0.09	-1.6 (-2.3 to -0.9)	<0.001	2.5 (1.1 to 3.9)	<0.001
Step change 4	-19.2 (-34.4 to -4.0)	0.01	14.7 (4.9 to 24.5)	0.003	-33.9 (-51.4 to -16.4)	<0.001
Slope change 4	-3.9 (-5.1 to -2.7)	<0.001	-0.8 (-1.6 to 0.1)	0.07	-3.1 (-4.6 to -1.6)	<0.001
**Opioid dependence**						
Step change 3	-1.7 (-20.4 to 17.1)	0.86	2.3 (-11.0 to 15.6)	0.73	-4.0 (-29.8 to 21.8)	0.76
Slope change 3	2.3 (1.1 to 3.4)	<0.001	-1.7 (-2.4 to -0.9)	<0.001	3.9 (2.4 to 5.4)	<0.001
Step change 4	-27.4 (-40.8 to -14.0)	<0.001	13.9 (4.2 to 23.7)	0.005	-41.3 (-57.6 to -25.1)	<0.001
Slope change 4	-4.7 (-5.9 to -3.6)	<0.001	-0.7 (-1.6 to 0.2)	0.13	-4.1 (-5.6 to -2.5)	<0.001
**Domestic violence and abuse**						
***Females***						
Step change 3	-5.9 (-19.8 to 8.0)	0.41	-3.3 (-13.2 to 6.6)	0.51	-2.6 (-21.1 to 15.9)	0.78
Slope change 3	0.2 (-0.8 to 1.1)	0.69	-1.8 (-2.4 to -1.2)	<0.001	2.0 (0.8 to 3.1)	0.001
Step change 4	-9.5 (-23.1 to 4.1)	0.17	13.8 (5.1 to 22.4)	0.002	-23.2 (-38.4 to -8.0)	0.003
Slope change 4	-2.6 (-3.6 to -1.6)	<0.001	-1.0 (-1.7 to -0.2)	0.01	-1.6 (-2.8 to -0.4)	0.01
***Males***						
Step change 3	2.6 (-7.5 to 12.8)	0.61	-3.7 (-9.5 to 2.1)	0.21	6.4 (-5.4 to 18.1)	0.29
Slope change 3	-0.7 (-1.4 to 0.1)	0.10	-1.1 (-1.5 to -0.8)	<0.001	0.5 (-0.4 to 1.3)	0.27
Step change 4	5.7 (-5.4 to 16.8)	0.31	7.3 (2.5 to 12.1)	0.003	-1.6 (-12.8 to 9.6)	0.78
Slope change 4	-0.5 (-1.5 to 0.5)	0.34	-0.7 (-1.1 to -0.3)	0.002	0.2 (-0.8 to 1.2)	0.70
**Intellectual disability**						
***Aged <14 years***						
Step change 3	-3.2 (-11.5 to 5.0)	0.44	-3.3 (-8.2 to 1.7)	0.20	0.0 (-9.5 to 9.5)	1.00
Slope change 3	-1.9 (-2.6 to -1.3)	<0.001	-1.2 (-1.7 to -0.8)	<0.001	-0.7 (-1.5 to 0.1)	0.08
Step change 4	17.2 (6.5 to 27.9)	0.002	18.4 (10.4 to 26.3)	<0.001	-1.2 (-13.3 to 10.9)	0.85
Slope change 4	0.2 (-0.8 to 1.1)	0.71	0.2 (-0.5 to 0.8)	0.59	0.0 (-1.1 to 1.1)	0.99
***Aged* ≥*14 years***						
Step change 3	-4.0 (-22.6 to 14.5)	0.67	-2.2 (-11.8 to 7.4)	0.66	-1.9 (-23.7 to 20.0)	0.87
Slope change 3	-2.3 (-3.7 to -0.9)	0.001	-1.6 (-2.1 to -1.0)	<0.001	-0.8 (-2.3 to 0.8)	0.34
Step change 4	-32.0 (-50.9 to -13.2)	0.001	8.4 (1.6 to 15.1)	0.002	-40.4 (-59.9 to -20.9)	<0.001
Slope change 4	-2.2 (-3.6 to -0.8)	0.002	-0.9 (-1.5 to -0.3)	<0.001	-1.3 (-2.8 to 0.3)	0.11
**Children with safeguarding concerns**						
Step change 3	-2.7 (-11.7 to 6.4)	0.56	-2.6 (-7.5 to 2.4)	0.31	-0.1 (-10.4 to 10.3)	0.99
Slope change 3	-1.5 (-2.3 to -0.8)	<0.001	-1.3 (-1.7 to -0.9)	<0.001	-0.3 (-1.1 to 0.6)	0.54
Step change 4	17.9 (5.7 to 30.2)	0.004	17.8 (10.1 to 25.6)	<0.001	0.1 (-13.2 to 13.4)	0.99
Slope change 4	0.1 (-0.9 to 1.2)	0.84	0.1 (-0.5 to 0.7)	0.74	0.0 (-1.1 to 1.2)	0.99

Step change 3: change in GP contact rate at the start of the second lockdown (05 November 2020). Slope change 3: change in slope of GP contact rate trend between combined second and third lockdown period (05 November 2020–28 March 2021) versus the period before the second lockdown (11 May 2020–04 November 2020). Step change 4: change in GP contact rate at the end of the third lockdown (29 March 2021). Slope change 4: change in slope of GP contact rate trend between the period after the third lockdown (29 March 2021–26 September 2021) and the period during the second and third lockdowns (05 November 2020–28 March 2021). RD = rate difference

## Discussion

### Summary

Pre-pandemic rates of primary care contact for vulnerable groups were more frequent than the general population, except for children with safeguarding concerns who had lower contact rates than their equivalent general population. All patients experienced a significant drop in primary care contact at the start of the first lockdown, and for those groups with alcohol misuse, those aged ≥14 years with intellectual disability, and children with safeguarding concerns, this drop was significantly larger than their general population comparators. Rates then steadily increased, with timings differing slightly between groups, and by the end of final lockdown, most general populations and vulnerable groups had contact rates exceeding those of before the first lockdown (with increases more marked in the vulnerable populations). For vulnerable children and their general population counterparts, post-pandemic contact rates remained lower than pre-pandemic rates.

### Strengths and limitations

This study used a large, nationally representative^19^ sample of 24 million patients (with the least populous vulnerable group comprising more than 8000 patients) and focused on vulnerable groups who have not previously been explored in this context. The CITS approach, with weekly-level temporal resolution, is a useful method for evaluating the effects of natural experiments on health outcomes.^27^

A potential limitation is that contact rates may not be reliably reported. For example, cancelled appointments may sometimes still be counted, and *ad hoc* patient contact, such as an unscheduled phone call to a patient, may not be recorded. However, the pre-pandemic rate of 5–6 contacts with primary care per patient per year in our study population is similar to the rates reported in other studies (around 4–5 contacts per patient per year).^14–16,28^ The issue with cancelled appointments may be more problematic in some vulnerable groups if they are more likely to cancel appointments than others; unfortunately we were unable to explore this further using the OpenSAFELY platform. The dynamic 1-year lookback period to define vulnerable groups may underestimate some cohorts, such as intellectual disability, where codes may not be continually recorded in primary care records following initial diagnosis. However, given that these issues, along with any other confounding issues, are likely to be reasonably constant over time, they should not have interfered with our analyses.

Primary care delivery and factors affecting access to care both changed during the pandemic in response to the pandemic itself and the lockdowns, which may have influenced the way data were recorded. The extent this impacted on results in unknown; however, we did not observe evidence of such non-lockdown changes affecting either vulnerable or general populations.

### Comparison with existing literature

Several studies documented changes in care during the first UK lockdown, including decreases in hospital admissions,^5^ the extent of disruption experienced by patients,^29^ and the rapid shift to online consultations.^10^ In the primary care context, studies of lockdown-related impacts are broadly consistent with our observed decreases in contact. Mansfield *et al* ^12^ reported the odds ratios for post- versus pre-lockdown contact rates ranging from 0.98 for acute alcohol-related events to 0.35 for diabetic emergency, while Murphy *et al* reported an 11% decrease in GP consultations between April and July 2020,^10^ compared with the same period the previous year.

A potential knock-on effect of reduced contact is reduced diagnosis rates (for example Williams *et al* ^13^ estimated decreases in diagnosis rates ranging from 16% for cancer to 50% for common mental health conditions during the first lockdown), which could lead to higher morbidity and mortality.

Attempts were made to address anticipated reductions in contact in some vulnerable groups. For example, telephone consultation availability was increased for patients with issues of drug misuse or dependence, and enhanced outreach services enabled continued service provision, albeit modified.^30^ Some opioid substitution therapy programmes continued through the lockdowns with changes to therapy provision.^30^ These may explain the reduced differential impact of the first lockdown on these groups.

There was evidence of increased DVA incidence under lockdown conditions^31^ and reduced referrals from GPs to the Identification and Referral to Improve Safety (IRIS) programme.^32^ The shift to remote consultations created challenges for GPs to provide DVA support; in response, practices implemented adaptations around DVA support messaging along with integrated approaches to identifying potential DVA cases.^31^

### Implications for research and practice

While the initial decreased contact for all groups was likely to be largely attributable to changes in how primary care was provided and accessed, along with government messaging to stay home and reduced infectious disease circulation, there are likely multiple drivers of the differential effects observed across vulnerable groups. As part of the rapid adaptation of primary care service provision with the first lockdown, there was a general shift towards prioritising the highest need patients,^10,33^ but the extent to which this prioritisation was proactive by healthcare providers or reactive based on patient demands is unclear and potentially varied widely.

Our findings indicate evidence of differential impacts of the UK COVID-19-related lockdowns on primary care contact for vulnerable groups. While the largest impacts were observed at the start of the first lockdown, evidence of differential impact persisted through subsequent lockdowns and beyond for some groups, with the most pronounced changes in the later phase occurring around end of the third lockdown. This study lays the foundation for exploring factors driving differences in primary care access and provision, not just during the pandemic but also examining inequities that may have persisted since.

## Data

All data used in this study were accessed via the OpenSAFELY platform. All analysis code and primary care codelists used to perform the analyses are publicly available online: https://github.com/opensafely/lockdown-and-vulnerable-groups
